# Frequency and Predictors of 30-Day Surgical Site Complications in Autologous Breast Reconstruction Surgery

**DOI:** 10.29252/wjps.8.2.200

**Published:** 2019-05

**Authors:** Hossein Masoomi, Berry Fairchild, Erik S. Marques

**Affiliations:** University of Texas Health Science Center at Houston, Division of Plastic and Reconstructive Surgery, Houston, Texas, USA

**Keywords:** Frequency, Predictor, Surgical site, Complication, Autologous, Breast, Reconstruction

## Abstract

**BACKGROUND:**

Surgical site complication (SSC) is one of the known complications following autologous breast reconstruction. The aim of this study was to evaluate the frequency and predictors of 30-day surgical site complications in autologous breast reconstruction.

**METHODS:**

American College of Surgeons National Surgery Quality Improvement Project (ACS-NSQIP) database was used to identify patients who underwent autologous breast reconstruction during 2011-2015. Multivariate regression analysis was performed to identify independent perioperative risk factors of SSC.

**RESULTS:**

Totally, 7,257 patients who underwent autologous breast reconstruction surgery were identified. The majority of the procedures were free flap (60%) versus pedicled flap (40%). The mean age was 51 years and the majority of patients were classified as American Society of Anesthesiologists (ASA)-II (60%) and 15% of patients had BMI>35. The overall 30-day SSC rate was 6.3%. The overall frequency of different types of SSC were superficial incisional infection (3.2%), wound dehiscence (1.8%), deep incisional infection (1.4%) and organ space infection (0.6%). BMI>35 (adjusted odds ratio [AOR]=2.38), smoking (AOR=2.0), diabetes mellitus (AOR=1.67) and hypertension (AOR=1.38) were significant risk factors of SSC. There was no association with age, ASA classification, steroid use, or reconstruction type.

**CONCLUSION:**

The rate of 30-day SSC in autologous breast reconstruction was noticeable. The strongest independent risk factor for SSC in autologous breast reconstruction was BMI>35. The type of autologous breast reconstruction was not a predictive risk factor for SSC. Plastic surgeons should inform patients about their risk for SSC and optimizing these risk factors to minimize the rate of surgical site complications.

## INTRODUCTION

Breast cancer is the most common cancer in woman.^1^ More than 230,000 new cases of invasive breast cancer were diagnosed in the United States in 2016.^2 ^Up to 40% of patients undergo reconstruction after mastectomy with rates of reconstruction increasing by approximately 5% per year.^3^^-^^5^ Autologous tissue transfer is frequently esthetically superior to alternative reconstructive options and often regarded as the optimal method of breast reconstruction.^6^

Surgical site complication (SSC) can be costly and associated with a delay in adjuvant therapy in the breast cancer patient. Unfortunately, wound complications impact up to half of patients following autologous breast reconstruction.^7^^-^^9^ Surgical outcomes rely on understanding and accounting for comorbidities that affect results. With an increasing number of women undergoing breast reconstruction, understanding risk becomes increasingly important. Reconstruction type can influence morbidity and patient satisfaction; therefore, discussion between surgeon and patient to determine best reconstructive options should include a thorough risk assessment.^11^^,^^12^

Numerous studies have evaluated risk factors of postoperative complications in autologous breast reconstruction and identified risks specific to certain comorbid conditions but are limited regarding predictor risk factors of SSC.^7^^,^^13^^,^^14^ The main purpose of our study was to evaluate the rate of SSC following free and pedicled autologous breast reconstruction using the American College of Surgeons- National Surgical Quality Improvement Program (ACS-NSQIP) database. Additionally, potentially modifiable risk factors of SSC after autologous breast reconstruction were identified with the goals of (i) to decrease wound complications and improve patient outcomes and satisfaction, (ii) to mitigate delays in adjuvant therapy for post-oncologic reconstructions and (iii) to decrease burden of health care costs. 

## MATERIALS AND METHODS

The American College of Surgeons-National Surgical Quality Improvement Program (ACS-NSQIP) database was a risk adjusted, and surgical outcomes-based program designed to measure and improve the quality of surgical care.^15^ Trained clinical reviewers prospectively collected the ACS-NSQIP data and validated them from medical records on preoperative risk factors, preoperative laboratory values, intraoperative variables, 30-day postoperative mortality, and 30-day morbidity on all patients undergoing major surgeries at participating institutions. The ACS-NSQIP database provided prospective national data with a large sample size making it ideal for identifying important differences in patient risk, as in 2015, the ACS-NSQIP database contained patients’ data for more than 885,502 cases from 603 participating hospitals. 

Using the ACS-NSQIP database, discharge data for female breast cancer patients who underwent free or pedicled flap autologous breast reconstruction surgery from 2011 to 2015 were analyzed. Current Procedural Terminology (CPT) codes of 19361 (breast reconstruction with latissimus dorsi flap, without prosthetic implant), 19364 (breast reconstruction with free flap), 19367 (breast reconstruction with transverse rectus abdominis myocutaneous flap -TRAM-, single pedicle), 19368 (breast reconstruction with TRAM flap, single pedicle with microvascular anastomosis), 19369 (breast reconstruction with TRAM flap, double pedicle) were used to identify the patient population. 

The main outcome was SSC which was defined by the presence of any of the following complications within the 30-days postoperative period including (i) superficial incisional infection, (ii) deep incisional infection, (iii) organ space infection or (iv) wound dehiscence. Diagnosis of SSC was made by the surgeon or attending physician. As defined by the ACS-NSQIP database, 30-day surgical site complications included (1) Superficial incisional infection as an infection that involved only the skin or subcutaneous tissue of the incision. (2) Deep incisional infection was an infection that involved the deep soft tissues (e.g., fascial and muscle layers) of the incision. 

(3) Organ space infection was an infection that involved any part of the anatomy (e.g., organs or spaces), other than the incision, which was opened or manipulated during an operation. An infection that involved both superficial and deep incision sites was reported as a deep incisional infection. Also, an organ space infection that drained through the incision was reported as a deep incisional infection. (4) Wound dehiscence referred primarily to loss of the integrity of fascial closure or whatever closure was performed in the absence of fascial closure.^16^ The overall rate of SSC and its subcategory specific to the type of autologous breast reconstruction were discussed. Perioperative factors that were analyzed included patients’ characteristics and comorbidities, smoking status, steroid use, the American Society of Anesthesiologists (ASA) classification, body mass index (BMI) and reconstruction type (pedicled vs. free). 

Univariate and multivariate regression analysis were performed to identify independent predictors of SSC following autologous breast reconstruction. Factors were statistically significant in univariate analyses or factors which clinically expected to have impact on SSC were included in multivariate regression analysis. The adjusted odds ratio (AOR) was calculated in multivariate regression analysis to determine the combined effect of various perioperative factors (age, patient comorbidities, smoking, steroid use, the ASA classification, BMI and reconstruction type) on SSC. All statistical analyses for the ACS-NSQIP data were conducted using Statistical Package for the Social Sciences (SPSS) statistical software (Version 21, Chicago, IL, USA). Statistical significance was set at p-values <0.05. AOR> 1 was considered the risk factor for free flap failure.

## RESULTS

A total of 7,257 patients underwent autologous breast reconstruction in this database during 2011-2015. Examining patients’ characteristics, the mean age was 51±10 years and 7.7% of them were older than 65 years. The majority of reported race was Caucasian (68.2%), followed by African American (11.8%). The most common comorbidities were hypertension (25.4%), smoking (9.4%) and diabetes mellitus (6.2%). The majority of the patients were classified in ASA-II (60%) and ASA-III (35%). With regard to BMI, 15% of patients had a BMI of 35 or higher (Table 1). 

**Table 1:** Characteristics of patients who underwent autologous breast reconstruction (ACS-NSQIP Database, 2011-2015)

**Variable**	**Overall**
**Characteristics**	
Number	7,257
Female	99.7%
Age (year)	
Mean	51.4±9.7
Median	52
Mode	52
Over 65 (%)	7.7
Race	
White	68.2
African American	11.8
Asian	2.9
Native Hawaiian	0.2
American Indian or Alaska Native	0.5
Unknown/Not reported	16.4
Comorbidity	
Diabetes mellitus	6.3
Hypertension	25.4
Congestive heart failure	0.0
Peripheral vascular disease	0.0
Chronic obstructive pulmonary disease	0.8
Chronic kidney disease on dialysis	0.1
Smoker*	9.4
Steroid use	1.8
The American Society of Anesthesiologists (ASA) classification	
1	5.1
2	59.8
3	34.9
4	0.2
Body mass index (BMI) classification	
BMI≥35	15.0

* Current smoker within one year

The majority of the autologous breast reconstruction was free flap reconstruction (60%). The mean length of hospital stay was 4.0 days. The overall 30-day SSC rate was 6.3%. The overall frequency of SSC subtypes were superficial incisional infection (3.2%), wound dehiscence (1.8%), deep incisional infection (1.4%) and organ space infection (0.6%) (Table 2 and 3). There was no significant difference observed in overall SSC rate in pedicled (6.7%) versus free flaps (6.1%, *p*=0.27). Evaluating SSC subtypes, there was no significant difference observed in superficial and deep surgical site infections and wound dehiscence in free versus pedicled flaps breast reconstruction except organ space infection which was significantly higher in pedicled flap (1.0%) when compared with free flap breast reconstructions (0.3%, *p*<0.01).

**Table 2:** Surgical characteristics and outcomes

**Variable**	**Frequency**
Characteristic	%
Autologous breast reconstruction type	
Free flap	60
Pedicled flap	40
Surgical site complication	
Superficial incisional infection	3.2
Deep incisional infection	1.4
Organ space infection	0.6
Wound dehiscence	1.8
Overall	6.3

**Table 3:** Frequency of surgical site complications in pedicled vs. free autologous breast reconstruction

**Surgical site complications**	**Pedicled flap ** **(%)**	**Free flap ** **(%)**	***p*** ** value **
Superficial incisional infection	3.2	3.3	0.92
Deep incisional infection	1.3	1.5	0.58
Organ space infection	1.0	0.3	<0.01
Wound dehiscence	2.0	1.6	0.28
Overall	6.7	6.1	0.27

Table 4 and Table 5 show the univariate and multivariate regression analyses for factors associated with higher rate of SSC in autologous breast reconstruction Multivariate regression analysis showed that BMI>35 (AOR=2.38, CI [Confidence Interval]=1.91-2.96; *p*<0.01), smoking (AOR=2.0, CI=1.52-2.58; *p*<0.01), diabetes mellitus ([DM] AOR=1.67, CI=1.21-2.28; *p*<0.01) and hypertension (AOR=1.38, CI=1.12-1.71; *p*<0.01) were significant risk factors of SSC. There was no association with age, ASA classification, steroid use, chronic obstructive pulmonary disease or reconstruction type (pedicled vs. free flap) on SSC (Table 5).

**Table 4:** Univariate regression analyses for 30-day surgical site complications in patients who underwent autologous breast reconstructive (NSQIP 2011-2015)

**Variable**	**AOR (95% CI** * **)**	***p*** ** value**
Age group		
<65 years	Reference	Reference
≥65 years	1.08 (0.75–1.55)	0.70
ASA Class	NS	
Comorbidities		
No comorbidities	Reference	Reference
Hypertension	1.70 (1.39–2.07)	<0.01
Diabetes mellitus	2.30 (1.70–3.10)	<0.01
Steroid use	1.36 (0.73–2.54)	0.34
Chronic obstructive pulmonary disease	2.71 (1.32–5.54)	0.02
BMI>=35	2.70 (2.18–3.32)	<0.01
Smoking	2.0 (1.52–2.60)	<0.01
Preoperative weight loss >10%	1.65 (0.38–7.14)	0.50
Reconstruction type		
Free flap	Reference	Reference
Pedicled flap	0.99 (0.81–1.21)	0.93

* AOR, Adjusted Odds Ratio; CI: Confidence Interval; NS: Not Significant

**Table 5:** Multivariate regression analysis for 30-day surgical site complications in patients who underwent autologous breast reconstructive (NSQIP 2011-2015)

**Variable**	**AOR (95% CI** * **)**	***p*** ** value**
Age group		
<65 years	Reference	Reference
≥65 years	1.17 (0.80–1.70)	0.42
Comorbidities		
No comorbidities	Reference	Reference
Hypertension	1.38 (1.12–1.71)	<0.01
Diabetes mellitus	1.67 (1.21–2.28)	<0.01
Steroid use	1.21 (0.64–2.29)	0.55
Chronic obstructive pulmonary disease	2.01 (0.98–4.37)	0.06
BMI>=35	2.38 (1.91–2.96)	<0.01
Smoking	2.0 (1.52–2.58)	<0.01
Reconstruction type		
Free flap	Reference	Reference
Pedicled flap	1.0 (0.91–1.11)	0.86

* AOR: Adjusted Odds Ratio; CI: Confidence Interval

## DISCUSSION

Surgical outcomes rely on understanding and accounting for comorbidities that affect results. With an increasing number of women undergoing breast reconstruction, understanding risk becomes increasingly important. This study, using the ACS-NSQIP database with a large number of patients, demonstrated the rate of 30-day SSC after autologous breast reconstruction surgeries to be relatively low (6.3%). However, the rate of surgical site complications in autologous breast reconstruction has been reported in previous studies as high as 49%.^7^^-^^10^

Also, we identified factors that increased morbidity following autologous breast reconstruction. Significant SSC risk factors, derived from regression analysis, included the following patient co-morbidities of BMI>35, smoking, diabetes mellitus and hypertension. In contrast, patient’s characteristics or type of breast reconstruction were not found to be significant risk factors for SSC. The strongest independent risk factor for SSC in autologous breast reconstruction was BMI exceeding 35 kg/m^2^ (AOR=2.38). Previous studies have demonstrated that obesity was associated with higher morbidity following a variety of surgical interventions including breast reconstruction and was a reliable predictor of wound complications following breast reconstruction.^17^^-^^19^

Currently, one-third of adults are obese. Accordingly, more obese individuals are seeking breast reconstruction, increasing the importance of identifying and understanding this patient risk factor.^20^^-^^22^ In a study evaluating 639 patients who underwent deep inferior epigastric perforator (DIEP) flap breast reconstruction, Ochoa *et al.*^9^ showed that increasing BMI predisposed patients to delayed wound healing complications in both flap and donor-site locations. Nevertheless, overall flap complications remained similar across all BMI groups.

A potential etiology of increased risk of wound complications in obese adults may be related to increased cardiac workload and impaired diaphragmatic descent secondary to large volume of adiposity resulting in decreased oxygenated blood flow to tissues. Subsequently without adequate oxygenation, fibroblasts cannot form collagen and tissue repair processes are impaired. Additionally, compromised vascularity impairs delivery of neutrophils, macrophages and other cells which aid in wound healing. Finally, habitus-related decreased mobility increases hygiene-related complications.^22^^,^^23^ Therefore, these patients should be informed regarding the higher risk of surgical site complications preoperatively.

Although COPD was found to be a risk factor of SSC in univariate regression analysis, this was not consistent based on multivariate regression analysis. Not surprisingly, diabetes mellitus (AOR=1.79) was one of the main risk factors of SSC. Diabetes mellitus with poorly controlled blood sugars impairs cellular function and impedes all stages of wound healing.^21^^,^^23^^-^^25^ As expected, smoking was a significant risk factor for SSC (AOR=2.0). Proposed etiology of this effect included (i) nicotine-caused vascoconstriction and decreased cutaneous blood flow, (ii) accelerated tissue destruction through release of proteases and suppressing the immune response and (iii) impaired collagen production.^21^^,^^26^

The presence of any one of these co-morbidities are risk factors for poor wound healing and complications. Although steroids are known to have dermal effects that can impact wound healing including inhibition of fibroblast proliferation and decreased collagen production,^22 ^the current study did not demonstrate steroid use as a risk factor of SSC. This finding is consistent with the study of Fischer *et al.*^4^ using the same database. Regarding the type of breast reconstruction (pedicled versus free flap), interestingly, we did not observe an overall significant difference in SSC rate between the two groups. 

However, we did note a statistically significant increase in organ space infections in the pedicled flap reconstruction (1.0%) versus free flap reconstruction (0.3%) group. Previous studies have shown a decrease in abdominal morbidity with use of various types of free flaps and touted the advantages of free flap including improved perfusion and decreased donor site morbidity, which may explain the observed differences in organ space-type infections.^17^^.^^27^ Overall, our results are congruent with data available suggesting that procedure type has no significant effect on complication rates.^11^

Regarding patient characteristics, advanced age was recognized as a risk factor for cancer. Moreover, the increase in life expectancy has increased the number of elderly patients who require surgeries for oncologic resections and seek reconstructions. It is important to evaluate the effect of advanced age in the outcomes of autologous breast reconstruction. Interestingly, Butz *et al.*^28^ in a study comparing the outcomes of breast reconstruction in elderly (>=65 years) patients with younger women, showed that there were no differences in the adjusted complication rates between older and younger patients undergoing implant-based reconstruction. 

Our study showed that advanced age was not a predictive risk factor for SSC. Conversely, Matsumoto *et al.*^10^ in a retrospective study evaluating the effect of advanced age in breast reconstruction, demonstrated that patients older than 60 years had a 1.6 times greater chance of surgical wound complication than the younger patients. There are several limitations to this study similar to retrospective studies using a large database. First, The ACS-NSQIP database is limited to 30-day outcomes, so data outside of this time period is not collected. This may underestimate the overall rate of SSC. 

Secondly, because procedure types are not evenly distributed across surgeons or centers, complication rates may be influenced by individual provider practice differences. Thirdly, we were unable to determine if the SSC was related to the donor or recipient site. Fourthly, the use of perioperative antibiotics in this patient population was unknown, which could be an important factor preventing surgical site infections. Finally, we were unable to evaluate the effect of chemotherapy and radiation in SSC as the majority of data were missing in this patient population. Despite the mentioned limitations, to our knowledge, this is the largest study concentrating on the risk factors of SSC in autologous breast reconstruction. 

In conclusion, this study characterizes the incidence of surgical site complications within 30-days following autologous breast reconstruction (6.3%). Identifiable SSC risk factors include obesity (BMI>35), smoking, diabetes mellitus and hypertension. We found that the type of reconstruction was less influential on postoperative wound complications. Plastic surgeons should inform patients about their risk for SSC and optimizing these risk factors that might help to minimize the rate of surgical site complications. Future prospective studies will be required to evaluate the risk factors of SSC in autologous breast reconstruction in detail.

## CONFLICT OF INTEREST

The authors declare no conflict of interest.
